# Genomic analysis of the emergence of 20th century epidemic dysentery

**DOI:** 10.1186/1471-2164-15-355

**Published:** 2014-05-10

**Authors:** Laurence Rohmer, Michael A Jacobs, Mitchell J Brittnacher, Christine Fong, Hillary S Hayden, Didier Hocquet, Eli J Weiss, Matthew Radey, Yves Germani, Kaisar Ali Talukder, Anthony J Hager, John M Kemner, Elizabeth H Sims-Day, Susana Matamouros, Kyle R Hager, Samuel I Miller

**Affiliations:** Department of Microbiology, University of Washington, Seattle, WA USA; Hygiène Hospitalière, University Hospital of Besançon, Besançon, France; UMR6249 Chrono-Environnement, Université de Franche-Comté, Besançon, France; Institut Pasteur, Paris, France; International Centre for Diarrheal Disease Research, Dhaka, Bangladesh; Department of Genome Sciences, University of Washington, Seattle, WA USA; Department of Medicine, University of Washington, Seattle, WA USA

**Keywords:** *Shigella dysenteriae*, Dysentery, Genome evolution, Phylogeny, Antibiotic resistance, Genomic adaptation, Human carrier, Pandemic

## Abstract

**Background:**

*Shigella dysenteriae* type 1 (*Sd*1) causes recurrent epidemics of dysentery associated with high mortality in many regions of the world. *Sd*1 infects humans at very low infectious doses (10 CFU), and treatment is complicated by the rapid emergence of antibiotic resistant *Sd*1 strains. *Sd*1 is only detected in the context of human infections, and the circumstances under which epidemics emerge and regress remain unknown.

**Results:**

Phylogenomic analyses of 56 isolates collected worldwide over the past 60 years indicate that the *Sd*1 clone responsible for the recent pandemics emerged at the turn of the 20^th^ century, and that the two world wars likely played a pivotal role for its dissemination. Several lineages remain ubiquitous and their phylogeny indicates several recent intercontinental transfers. Our comparative genomics analysis reveals that isolates responsible for separate outbreaks, though closely related to one another, have independently accumulated antibiotic resistance genes, suggesting that there is little or no selection to retain these genes in-between outbreaks. The genomes appear to be subjected to genetic drift that affects a number of functions currently used by diagnostic tools to identify *Sd*1, which could lead to the potential failure of such tools.

**Conclusions:**

Taken together, the *Sd*1 population structure and pattern of evolution suggest a recent emergence and a possible human carrier state that could play an important role in the epidemic pattern of infections of this human-specific pathogen. This analysis highlights the important role of whole-genome sequencing in studying pathogens for which epidemiological or laboratory investigations are particularly challenging.

**Electronic supplementary material:**

The online version of this article (doi: 10.1186/1471-2164-15-355) contains supplementary material, which is available to authorized users.

## Background

Dysentery caused by *Shigella dysenteriae* type 1 (*Sd*1) is a recurrent challenge in many parts of the world. Epidemics of this disease are associated with a high rate of mortality in young children [1]. Treatment is complicated by the rapid emergence of *Sd*1 strains resistant to the newest antibiotics [2]. No vaccine protective against *Sd*1 is currently available, but efforts to create one are underway [1, 3]. *Sd*1 was first identified in Japan at the end of the 19th century, during a pandemic that killed thousands [4, 5]. The most recent pandemics took place in Central America between 1968 and 1972, South Asia in the mid 1970s, Central Africa and South East Asia in the 1980s, and East Africa in the 1990s [3]. Intermittent outbreaks still hit these regions, such as Guatemala in 1991 [6] and Cameroon in 1998 [7]. Typically, deteriorated hygiene conditions and overcrowding contribute to the occurrence of outbreaks. The spread of *Sd*1 infection is correlated with human activity and population density rather than water, which has been associated with outbreaks of other types of *Shigella*[8]; however, the specific circumstances under which epidemics emerge are not understood. Between outbreaks, few sporadic cases, if any, are documented. Humans are the only known hosts, and no natural reservoir has ever been identified. *Sd*1 is transmitted through the fecal-oral route, by direct contact with an infected person or by contamination of food or surfaces [5].

Genomic analyses have revealed that *Sd*1 descends from an *Escherichia coli* strain that gained the ability to colonize the mucosal epithelium cells of the large intestine [9–11]. This phenotype is made possible by functions encoded on an invasion plasmid and on the chromosome, and it is enhanced by the loss of some functions inherited from the ancestral *E. coli*[12]. In addition to being the deadliest *Shigella* strain, *Sd*1 distinguishes itself from other *Shigella* by its atypical invasion plasmid, which is a combination of the two known variants, pINVA and pINVB [13], and by the production of the Stx1 toxin that is also produced by multiple virulent *E. coli. Sd*1 is the most infectious of all *Shigella* strains, causing disease with an inoculum as low as 10 CFU [14].

Studying *Sd*1 is made difficult by the fact that stool samples must be immediately and properly stored and refrigerated in order to recover viable *Sd*1 isolates [15]. *Sd*1 outbreaks often take place in already dire circumstances (e.g. war, natural disasters) in which its proper collection, immediate refrigeration and study is problematic. The investigation of *Sd*1 pathogenesis is also challenging due to the lack of an appropriate animal model. To gain some insight into this elusive pathogen, we investigated the genetic diversity and population structure of 56 strains collected in the regions that underwent the most recent pandemics. From whole-genome analysis it appears that the prominent *Sd*1 lineages emerged in the recent past and remained ubiquitous over the 20^th^ century. *Sd*1 genomes evolve with a relative high rate of substitution and substantial horizontal transfer, and mostly without selection. We explored the implication of this evolution for the management and diagnosis of future outbreaks.

## Results

### Genomic diversity of *Sd*1 strains involved in major pandemics

The genomes of 55 *Sd*1 strains were sequenced using Illumina NGS Technology (see Methods) and assembled *de novo* (Additional file 1). Strains originated from Central America [16], Africa [17–19] and Asia [20–22] (Additional file 1 and 2). This set of strains, collected from patients over the course of outbreaks or as sporadic cases in endemic regions, encompasses every region in which the main pandemics took place [3]. In addition, a strain collected in Tennessee from a child with no history of travel was sequenced. The publicly available genome of *Sd*197, a strain isolated in China in 1949 [22], was included in our analyses.

Multi-copy IS elements make up approximately 25% of *Sd*1 genomes and cause frequent contig breaks during the assembly process (Additional file 1). To assess genetic diversity among the 56 strains, we first created consistently annotated genomes and organized gene content by orthologous families using PGAT [23]. A total of 3,591 gene families were identified from the chromosome: 2,807 are present and functional in every strain (core genome); 784 are present or functional in only a subset of strains (accessory genome), including 237 that were lost (those missing in some *Sd*1 subclades but present in the closest *E. coli* genomes) and 547 gained (present in some *Sd*1 subclades but not in the *E. coli* relatives or inconsistently distributed across phylogenetic groups) (Additional file 3). Gene content is highly homogenous and gene order is highly conserved among strains, as seen in contigs large enough to reveal synteny (Additional file 1). Insertion sites of IS elements are also conserved suggesting that most insertions took place in the ancestor of all these *Sd*1 strains (Additional file 1). Because the invasion plasmid (pINV), required for *Sd*1 to cause disease [24], could not be fully assembled using our method (see Methods), we were not able to assess gene content or order; however, we investigated single nucleotide polymorphisms (SNPs) and found very limited variation in these strains (Additional file 4).

### *Sd*1 strains genetic relatedness and intercontinental distribution

**Figure 1:**
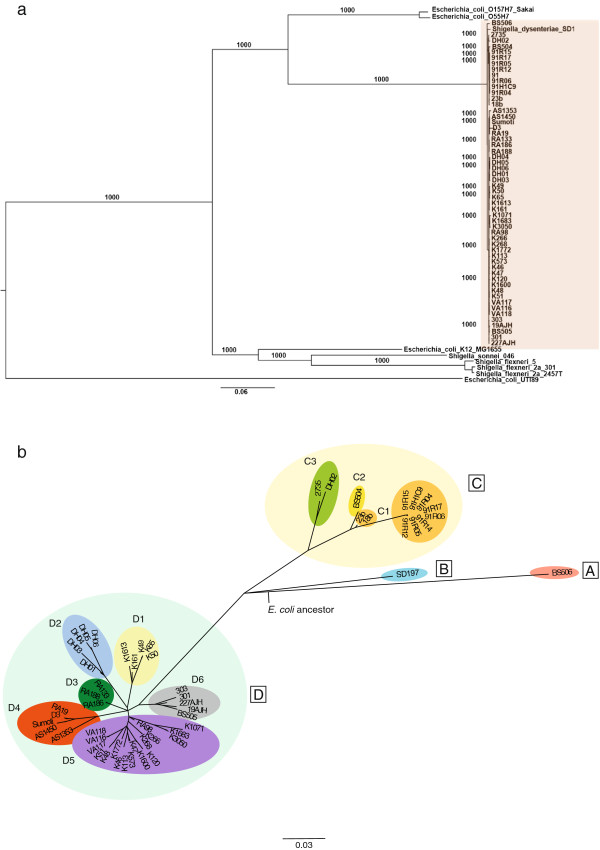
***Shigella dysenteriae*** **type 1 phylogeny. a**. Maximum likelihood phylogeny of the 56 *Shigella dysenteriae* type 1 strains, relative to *E. coli* K12, O157, O55, *Shigella flexneri* and *Shigella sonnei.* The root of the tree was determined using the more distant relative *E. coli* UTI89. The tree was constructed using 78,267 variable positions found over 1,859 genes with 1,000 Bootstrap repetitions (values displayed on the branches). *Sd*1 strains are framed in orange. A neighbor-joining tree based on the rate of synonymous substitutions (dS) between each pair showed the same topology as the maximum likelihood tree, with similar relative branch lengths (data not shown). **b**. Maximum likelihood phylogeny based on a concatenation of the 919 polymorphic positions identified between the 56 *Shigella dysenteriae* type 1 strains in the common genes that are single-copy and not subjected to recombination (see Methods). The root (*E. coli* ancestor) was approximately placed based on the *E. coli* outgroups (Figure 1a). Geographic distribution of lineages or sub-lineages: A. Tennessee, B. China, C1. Guatemala, C2. Zambia, C3. Cameroon and an unknown location, D1. Bangladesh, D2. Central African Republic, D3. Bangladesh, D4. Bangladesh and India, D5. Bangladesh and D6. India and Thailand.

To investigate the relatedness between the 56 *Sd*1 strains, we built a phylogenetic tree that included a subset of *E. coli* genomes and genomes of other *Shigella* species (Additional file 5). Using PGAT [23], we identified a set of 1,859 genes for which an ortholog was present as a single copy in every genome included in the phylogenetic analysis (Additional file 6). Nucleotide alignments were generated for each gene family from which a total 78,266 SNPs were extracted. The maximum likelihood tree in Figure 1a shows that all *Sd*1 strains (framed in orange) are closely related and form a clade separate from *E. coli* and other *Shigella* species. The overall tree topology is the same as previously published [11]. The branch leading to the *Sd*1 strains is longer than the others, suggesting that this lineage was subjected to a higher rate of substitution or a higher rate of gene recombination than its *E. coli* and *Shigella* relatives. Although recombination events do not influence the topology of the species phylogenetic tree [11, 25], recombination is known to take place in *E. coli* genomes, and to contribute to sequence divergence. Hence, to assess the role of recombination in the evolution of *Sd*1 ancestral genome, we estimated the number of SNPs in the alignment that were due to recombination for each genome (see Methods). For the 1,859 genes used to build the phylogeny, all *Shigella* genomes exhibit a higher rate of recombination compared to *E. coli* genomes (Additional file 7). However, the extent of recombination overall might be underestimated for the *E. coli* genomes because the genes identified as hot spots for recombination in *E. coli* are part of the accessory genome, hence not included in the set of 1,859 genes [25]. The number of SNPs predicted to be the result of recombination in *Shigella sonnei* and *Shigella flexneri* genomes is higher than the number of SNPs in the genome of *Shigella dysenteriae* (4,510, 6,308 and 3,011 respectively). This suggests that the greater length of the branch observed for *Sd*1 in the phylogenetic tree (Figure 1a) compared to the other branches in the tree is not only due to recombination events, but also to a higher substitution rate compared to the other *Shigella* subspecies and *E. coli*.

Phylogenetic relationships between *Sd*1 strains were reconstructed in more detail based on the 919 SNPs found within the *Sd*1 core genome (Methods). Only 689 genes out of the 2,807 *Sd*1 core genes were polymorphic across these strains due to mutations, and no trace of recombination could be found in the individual *Sd*1 strains, suggesting that no recombination event took place after their divergence from the common ancestor. The maximum likelihood tree (Figure 1b) shows that four lineages arose almost simultaneously from one unique ancestor. Lineage A is solely represented by BS506, a strain that was not associated with an outbreak (Tennessee, 1994) and lineage B by Sd197 (collected in China over the course of an epidemic, 1949). Lineages D and C co-exist in different parts of the world. For example, strains in lineage D are from India, Bangladesh, Thailand and Central African Republic, suggesting that strains moved around the world rapidly and recently.

### Recent emergence of the clone responsible for the latest pandemics and multiple intercontinental transfers

The paucity of SNPs and the lack of observed recombination events among *Sd*1 genomes suggest that the common ancestor to all these strains is very recent. Overall the genetic distance between the root and the *Sd*1 strains is consistent with the dates on which each strain was collected (R^2^ = 0.445, Additional file 8), providing a temporal signature of evolution. Using a Bayesian approach [26], we estimated the age of the most recent common *Sd*1 ancestor based on the concatenation of the 919 polymorphisms (Methods, Additional file 9). We selected the model that yielded the best AICM value [27]: the Gaussian Markov random field skyride model, assuming a lognormal relaxed clock and the GTR substitution model [28] (Methods, Additional file 10). This model allows for variation of the substitution rate across the different branches of the tree. The resulting mean substitution rate is 1.61E-03 substitutions per site per year, over the 919 polymorphic positions. The 919 polymorphic positions were detected across 2,807 core genes representing 2,270,268 bp (sum of the length of the 2,807 genes). Based on these sequences, the rate of substitution genome-wide is about 6.52E-07 (95% HDP: 4.61E-07 - 8.42E-07). This is slightly higher than *Shigella sonnei* for which a rate of 6.0 × 10^-07^ substitutions per site per year has been calculated [29]. According to this model, lineages C and D seem to have emerged around the 1940s and 1950s respectively (Additional file 10) and subsequently spread worldwide. The most recent common ancestor of lineages D1 (Bangladesh) and D2 (Central African Republic) dates from 1972 (95% HDP: 1960–1985), implying a recent intercontinental transfer. Similarly, the most recent common ancestor of strain C2 from Zambia and the strains collected in Guatemala likely dates from the beginning of the 1960s (1961, 95% HDP: 1952–1967), a mere eight years before the major outbreak that plagued Guatemala affecting over 100,000 people and killing more than 10,000 of them [6, 16]. This suggests another recent transfer from Africa to Central America, followed almost immediately by a vast clonal expansion. A rapid clonal expansion is also observed in all the subclades of lineage D, where the most recent common ancestors seem to pre-date the strains collected for the subclade by less than 10 years. The ancestor of all *Sd*1 strains in our collection spread across the world around the beginning of the 20^th^ century (1924 95% HDP: 1900–1942). Consistent with this timeframe, previous research on *Shigella* invasion plasmids concluded that *Sd*1 appeared more recently than the other *Shigella dysenteriae* serotypes and *Shigella* species [13]. It is conceivable that this *Sd*1 clone spread worldwide as an aftermath of World War I (1914–1918), a period of unusually high intercontinental transfer of people, troops and displaced populations, and conditions very favorable for outbreaks: high population density and poor hygiene conditions.

While no *Sd*1 cases were reported between the 1970s and 1990s in Guatemala, the clone that caused the 1991 outbreak is the direct descendant of the major 1970s outbreak (lineage C1). The lack of genetic diversity among the 1991 strains, which were collected at two different sites in Guatemala, indicates that only one clone was responsible for the entire 1991 outbreak (dated by BEAST from 1987, 95% HDP: 1984–1990). This clone has accumulated additional mutations compared to its close relatives collected in the 1970s, as illustrated by the relatively long branch in the tree (Figure 1b). Hence, it seems that *Sd*1 replicated at a high rate over 20 years despite no cases being reported. In lineage D5, the strain (RA98 - Bangladesh) dating from 1984 is substantially closer to the root than the other strains (dating from 2000s) and was collected during an outbreak, while the more recent strains were reported as sporadic cases. This data suggest that after an outbreak, the strain is maintained in the population and is only sporadically detected.

### Genetic drift responsible for most gene loss and mutations

**Figure 2:**
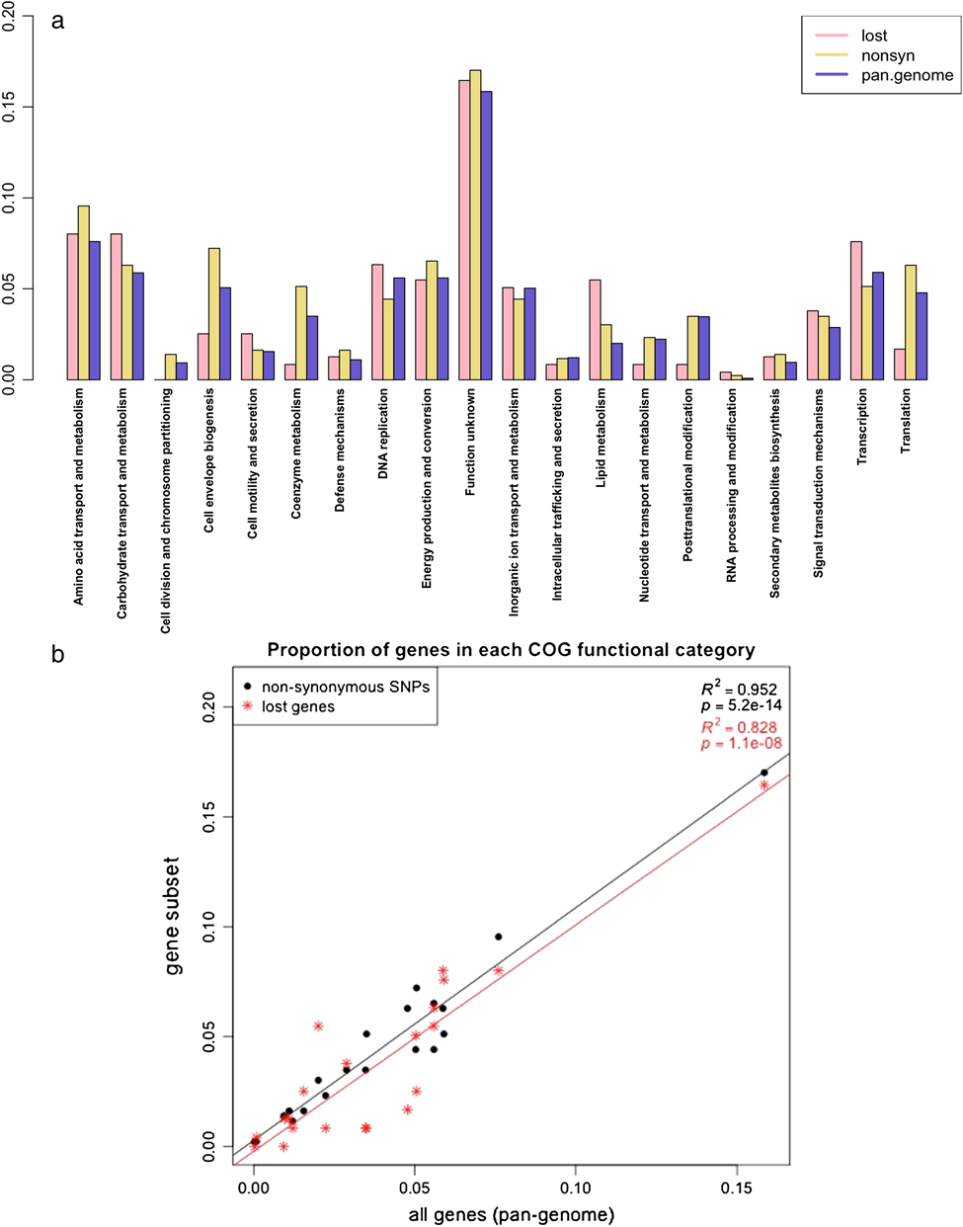
**Similarity of genes distributions across COG functional categories. a**. Distribution of genes in COG functional categories for the entire pan-genome, the genes that were lost in some strains and the genes modified by non-synonymous substitutions (expressed in percentage of genes in each group versus total number of genes). Genes for whom no function could be assigned were left out (lost: 17.18%, non-synonymous: 7.92%, pan-genome: 18.75%). **b**. Genes lost and genes modified with non-synonymous substitutions tend to distribute across functional categories in a similar fashion as genes overall (Pearson correlation = 0.9097 and 0.9758 respectively) indicating that the loss or modification of genes are selected at random. Only one functional category contains more lost genes than expected: lipid metabolism. In contrast, the functions of genes that were gained do not reflect the distribution of genes overall across functional categories (Pearson correlation = 0.5087) (data not shown).

Several scenarios could explain the observed predominance of two lineages in the most recent pandemics. Lineages with mutations or gene losses promoting fitness in the host (such as those conferring antibiotic resistance) may expand and replace previously existing clones. For example, a clone of *Salmonella enterica* Typhi carrying a mutation that confers resistance to fluoroquinolones has recently expanded within Southern Asia and may replace the existing clones there [30–32]. If mutations are selected for the advantage they confer or against their detrimental effect, the distribution of genes that are mutated or lost across functional categories is likely to be non-random. Alternatively, genes gained may favor clonal expansion and dissemination, as it has been observed with multiple drug resistance clones of *Shigella sonnei* and chloramphenicol-resistant *Salmonella enterica* serovar Typhimurium clones [29, 33]. In *Sd*1 genomes, the distribution of non-synonymous substitutions, found in 429 genes (Additional file 9), and most gene loss (Additional file 3) across functional categories is comparable to the distribution of all genes in the pan-genome across categories, R^2^ = 0.9523 and R^2^ = 0.8276 respectively (Figure 2a,b). This indicates that no group of function in particular is targeted by selection, and therefore *Sd*1 genomes seem to evolve mostly by genetic drift. The lack of selection on *Sd*1 genomes is further supported by the overall dN/dS ratio between each pair of genomes (non-synonymous substitution rate/synonymous substitution rate): all pairwise comparisons yield a ratio close to 1 (Additional file 8), indicating a random accumulation of mutations after the expansion of the original *Sd*1 clone.

Advantageous mutations are detected in a few subclades. For example, a mutation in ParC (S-80-I) [34, 35] which confers resistance to quinolones is found in subclade D4 (Bangladesh, and India, 1984 to 2003). Interestingly, although *Sd*1 cases were treated with fluoroquinolones in Bangladesh over that period of time [36], this resistant clone has not replaced the susceptible ones (D1, D3, D5 and D6). Furthermore, genes involved in lipid metabolism are lost at a higher rate than expected (Fisher exact test p-value = 0.0018). The inability to synthesize some lipids could alter the cell surface, perhaps hindering cell surface recognition by the host immune system [37]. Unfortunately, the impact on the cell surface of these losses cannot be determined solely based on the genes’ annotation.

**Figure 3:**
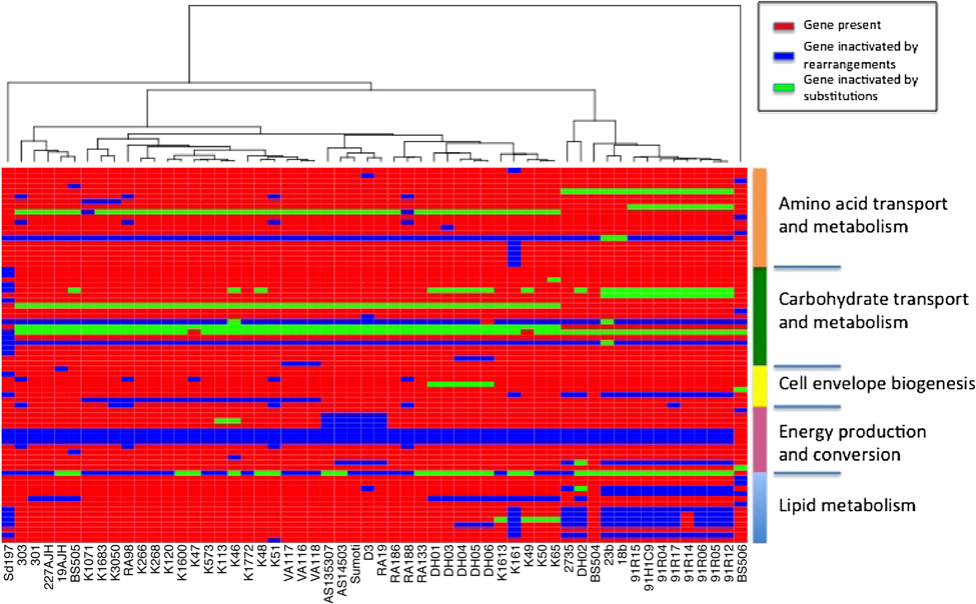
**Distribution across COG categories of the genes lost.** Genes that are lost in some strains for which a COG functional category is available are represented in this heatmap, color-coded by the type of genetic change resulting in the loss. The dendrogram is the topology of the maximum likelihood tree from Figure 1b.

The distribution of genes lost across the 56 strains (Figure 3) emphasizes the fact that genetic drift affects functions from all metabolic categories. As a consequence, phenotypes such as ability to metabolize some sugars or synthesize certain amino-acids may vary across strains. Since isolation and identification of bacterial strains often relies on this type of phenotypes, it is possible that in the future some diagnostic methods, used to detect *Sd*1 [15] may be compromised if they do not rely on additional components.

### Adaptation through gene gain: repeated acquisition of antibiotic resistance

**Figure 4:**
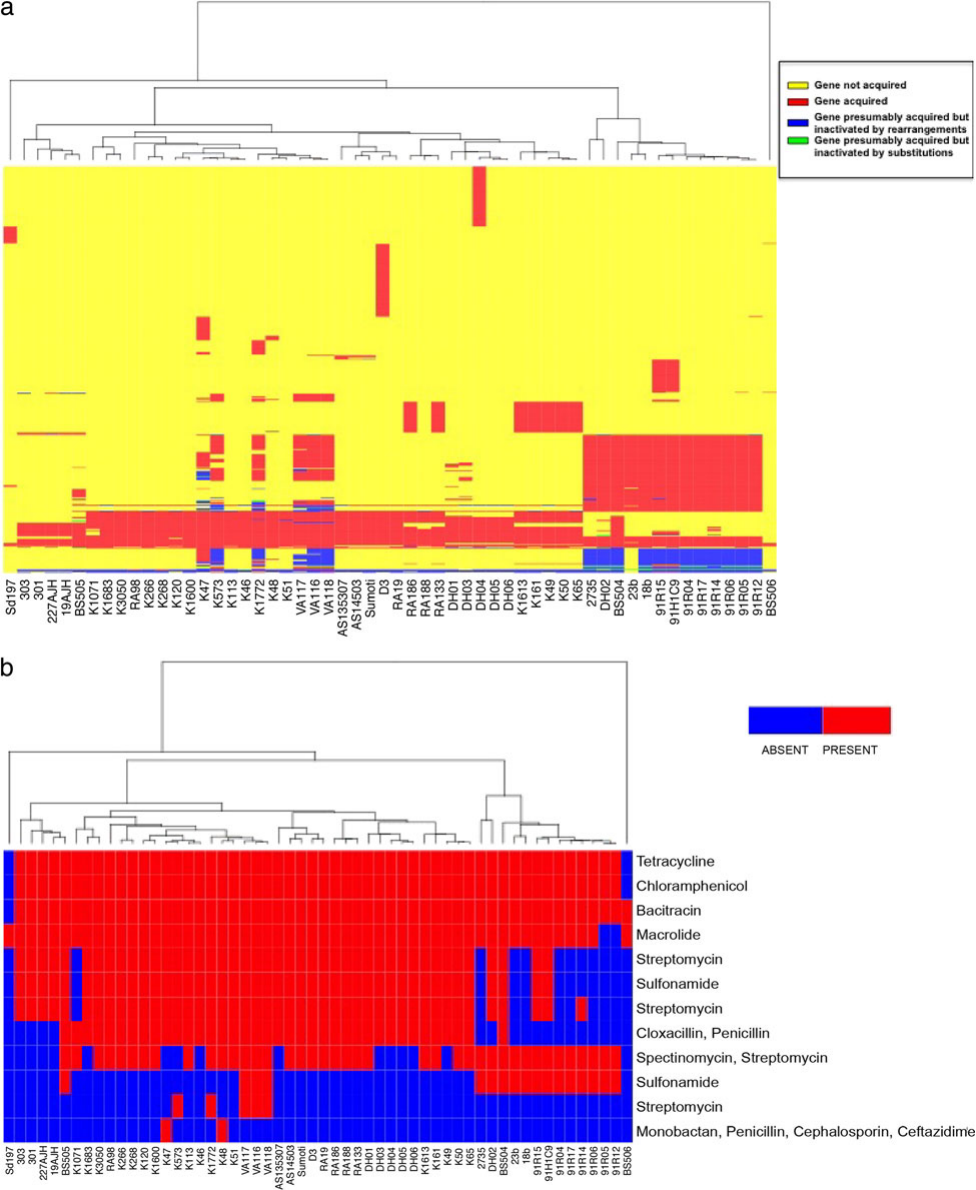
**Distribution across genomes of the genes gained over the course of** ***Sd*** **1 evolution. a**. Distribution of genes gained based on their presence and absence in the genomes contributing to the pan-genome. The dendrogram is the topology of the maximum likelihood tree from Figure 1b. **b**. Distribution across genomes of the acquired genes identified as conferring antibiotic resistance. Antibiotic resistance genes were identified by sequence comparison (blast) with the Antibiotic Resistance Database (ARDB). The dendrogram is the topology of the maximum likelihood tree from Figure 1b.

The 547 genes gained by one or more strains are carried on phages or plasmids (Additional file 3). In total, 199 genes with an analogous function were independently gained by strains that are evolutionarily distant from each other. Figure 4a illustrates the pattern of acquisition of these genes (y axis) across different lineages (x axis), and a description of all accessory genes and their distribution across strains can be found in Additional file 3, and their nucleotide sequences in Additional file 11. Approximately 33% have functions associated with mobile elements, such as *tra* genes, phage integrases, plasmid replication and partition (see below), while the others may bring new functions, such as antibiotic resistance. The observed independent and repeated gains suggest that these genes may confer a survival or fitness advantage. For example, a chloramphenicol resistance gene has been acquired at least five times in lineages C and D, and tetracycline resistance genes at least four. A gene cluster that confers resistance to tetracycline, and the transposon Tn21 carrying resistance genes to chloramphenicol, mercury and β-lactams (*bla*_OXA-1_) have been gained conjointly at least twice in lineage C, on two different plasmids. The plasmid in lineage C1 is very similar to R100 from *Shigella flexneri* 2b (NC_002134.1), while the plasmid in lineage C3 has an IncB backbone and resembles *Escherichia coli* HUSEC41 plasmid pHUSEC41-1 [38]. In lineage D, the genomes in subclades D1, D3, D4 and D5 also carry the tetracycline and chloramphenicol resistance genes. Those genes were found on an element very similar to a portion of the *Shigella* Resistance Locus pathogenicity island of the *Shigella flexneri* 2a [39] (AF326777.3). It is inserted at the Ser tRNA (codon UCC) locus. This element also contains CP4-associated prophage genes, a potential haemolysin, an anaerobic decarboxylate transporter and an aspartate racemase potentially involved in cell envelope/outer membrane biogenesis. Subclade D2 genomes also contain the antibiotic resistance genes, as well as some of the CP4 proteins, but lack the haemolysin. These genes in subclade D2 are carried on a plasmid of undetermined origin. Subclade D6, which diverged before the other subclades in lineage D, only carries the tetracycline resistance genes, but the assemblies did not unambiguously determine their positions in these genomes. Neither the strain from lineage A (BS506 from Tennessee), nor the strain from lineage B (Sd197, from China), contains any of these resistance genes. We identified additional antibiotic resistance genes (Figure 4b) whose distribution was also inconsistent with phylogeny. For example, three antibiotic resistance genes (*strA*, *strB*, *sulII*) are found in all but one strain of clade D. In these strains, these resistance genes are carried by a plasmid (pSFxv_3) previously identified in *Shigella flexneri* strain 2002017, an epidemic pathogen in China [40]. The same three genes are found in two strains from the 1991 Guatemala outbreak (91H1C9 and 91R15), on a different plasmid that is a composite of different plasmid pieces found in *Enterobacteriaceae*. Since the most recent common ancestor for subclade C1 is dated from 3 years prior to the outbreak, the acquisition of this mobile element occurred soon after the beginning of the outbreak, and was thereafter rapidly selected. Hence, it appears that *Sd*1 strains recurrently overcome antibiotic treatments through the acquisition by their genomes of mobile elements carrying resistance genes.

## Discussion

The *Sd*1 strains collected over the course of several outbreaks are genetically close to each other. They are distributed in four clades, of which two are prominent in regions where the latest pandemics broke out. These two clades appeared during the 1940s-1950s, while the most recent common ancestor for all the strains in our collection likely emerged in the 1920s. It is possible that the spread of these clones is the result of the two world wars of the 20^th^ century. The two conflicts provided ideal conditions for outbreaks and allowed for worldwide dissemination of *Sd*1 clones through massive population movements. Our data indicates that further intercontinental transmission occurred between Asia and Africa (in the 1950s for lineage D) and potentially between Africa and Central America (in the 1960s for lineage C). This lack of consistency between phylogeny and geography demonstrates the ability of *Sd*1 to be transferred from one continent to another and immediately cause outbreaks there. This could suggest that *Sd*1 has a different mode of transmission than some other bacterial pathogens causing pandemics, such as *Mycobacterium tuberculosis*[41], *Shigella sonnei*[29], *Neisseria meningitidis*[42] and *Yersinia pestis*[43], whose respective phylogenies are more consistent with their geographical distribution of isolates. Based on the branch lengths in the tree and the rate given by BEAST, *Sd*1 genomes sustain a relatively high rate of substitution compared to the genomes of *E. coli* and other *Shigella* species. The majority of substitutions observed in the two main clades do not appear to have been subjected to selection over the last 60 years. Genetic drift, an accelerated rate of mutation and lack of consistency between phylogeny and geography has already been observed for the enteric human-specific pathogen *Salmonella enterica* serovar Typhi [32]. This pattern has been attributed to this pathogen’s maintenance and transmission by asymptomatic chronic carriers [32]. It is tempting to speculate that similar to *Salmonella enterica* serovar Typhi, *Sd*1 could remain carried for a relatively long time by individuals that may not display the severe symptoms associated with disease, such as adults with significant protective immunity and/or resistance to severe disease. This could explain why sporadic cases of *Sd*1 are observed between outbreaks, such as in Bangladesh, and why *Sd*1 seemingly disappears from a region only to cause a new outbreak 20 years later, e.g., in Guatemala [6] or India [44]. Such a mode of transmission and maintenance would also explain the multiple intercontinental transfers, quickly followed by an outbreak. Fluoroquinolones are heavily used in South-East Asia to treat *Sd*1, and therefore constitute a strong selective pressure in the evolution of *Sd*1 in this region. But subclade D4 (fluoroquinolone resistant), which includes isolates from the 1980s and the 2000s, has not replaced the susceptible clones causing outbreaks in the region. This lack of clonal replacement is also consistent with the constraints associated with human asymptomatic carriers on the spread of *Sd*1. Long-term carriers of *Shigella*, including one *Sd*1 nearly asymptomatic carrier, have already been reported [45]. In this study, *Sd*1 could not be consistently detected in the feces of the carrier [45].

Acquisition of antibiotic resistance genes through horizontal transfer is frequent, as illustrated by the numerous independent gains of tetracycline and chloramphenicol resistance genes in lineages D and C, through different mechanisms. This suggests that although the genome evolution is neutral over the long-term, strains that carry the genes enabling them to defeat the antibiotic treatments are favored during an outbreak. In the case of a pathogen with a human carrier state, short-term selection and long-term neutrality are not mutually exclusive. Over the course of an outbreak, strains that acquire resistance genes may be rapidly selected, and appear overrepresented, but these are not necessarily the strains that will be maintained through the human carrier state [32]. This is especially plausible if the carrier did not exhibit the usual severe symptoms, in which case no selective pressure through antibiotic treatment would take place.

Genetic drift affects functions from all metabolic categories. This may result in a rapid divergence of biochemical phenotypes used by diagnostic tools: varying cell surface composition may interfere with serotyping and variation in sugar fermentation and amino-acid synthesis abilities may invalidate detection methods for *Sd*1 based on bacterial cell culture [15]. Hence, such methods may not be totally efficient at detecting *Sd*1 in individuals, whether they present symptoms or not. A combination of tools to monitor the presence of *Sd*1 may therefore be preferable to ensure that *Sd*1 is consistently detected and to prevent future outbreaks.

The ability of *Sd*1 to gain and retain genes and the possible existence of a carrier state may make it difficult to eradicate. It is likely that a combination of factors is responsible for the onset of outbreaks, such as a sudden deterioration of living conditions, caused by a war or a disaster, a change in a human carrier causing excretion of the pathogen, *e.g.* micronutrient deficiency or the disruption of the intestinal microbiome [46], or the interaction between the carrier of the *Sd*1 strain and an immunologically naive population.

Although no *Sd*1 outbreak has been reported since the early 2000s, we cannot rule out *Sd*1 re-emergence, particularly in the setting of war or famine. In conclusion, a comparative genomic analysis of a variety of strains from different locales over the last 60 years indicates a possible mechanism for epidemic emergence of this important human pathogen and suggests that comparative genomic approaches are particularly helpful to investigate pathogens whose lifecycle is elusive and for which no environmental reservoir is known.

## Conclusions

This study illustrates the important role of phylogenomic and comparative genomics analyses based on whole-genome sequencing for studying human-specific pathogens. Results of these analyses point to long-term human carriers as means of *Shigella dysenteriae* type 1’s maintenance and dissemination, and provide justification for a detailed epidemiological investigation, particularly where *Sd*1 has become endemic. Our analysis of the pan-genome suggests that the ongoing neutral evolution of *Sd*1 strains may result in rapid divergence of phenotypes used by diagnostic tools and provides data for the design of new tools, should the current ones become compromised. Next-generation sequencing technologies facilitate the investigation of pathogens that cannot be extensively studied in a laboratory or in the field, and help elucidate their biological lifecycle and their underlying epidemiology.

## Methods

### Bacterial strains, genome sequencing

The strains used in this work are described in Additional file 1. Genomic DNA was isolated by alkaline lysis, and was then sheared using a Biorupter UCD-200 (Diagenode Inc., Denville, NJ) and end-repaired. Repaired fragments were subjected to A-tailing using Taq DNA polymerase, and custom “Y” adaptors produced by hybridization of partially complimentary sequences were ligated to A-tailed fragments using T4 DNA ligase [47]. Paired-end libraries for each genome (insert size varying between 200 and 750 bp) were used to generate 76 bp or 100 bp reads with the Illumina GAIIx or Illumina HiSeq 2000 (coverage > 150 reads/genomic position). Sequencing of libraries was performed according to manufacturer’s standards (Illumina Inc. San Diego, CA). The resulting reads are summarized in Additional file 1 and are available through a bioproject (accession number PRJNA186649) at the National Center for Biotechnology Information (NCBI).

### Genome draft assembly and alignment

Reads were assembled with the Columbus module of Velvet software v1.1 [48] using the sequence of Sd197 as a reference (NC_007606, NC_007607 and NC_009344). Assemblies were corrected by realigning the reads onto the contigs with BWA and searching for discrepancies using SAMtools [49, 50]. Contigs were extended or joined based on consistent read mappings with their mates. Details for these assemblies are provided in Additional file 1. To estimate the percentage of the genome covered by the assembly, we aligned the draft genome to the Sd197 genome sequence using Nucmer [51] and compared the length of the reference sequence covered by the assembly with the length of the reference sequence covered by sequencing reads (aligned using BWA [49]). The invasion plasmid (pINV) was not assembled, due to low sequence read coverage and a large number of repeats in the plasmid sequence. SNPs in single copy protein coding genes on pINV were identified by aligning sequence reads for each strain onto the complete sequence of pINV from Sd197 and searching for discrepancies, as described for assembly correction above. Details for these SNPs are provided in Additional file 4. Seven genomes had too little coverage of pINV to determine variants.

### Sequence annotation

Genomes were annotated using PGAT as described previously [23]. Briefly, we grouped genes belonging to 75 *E. coli* and *Shigella* genomes by orthologous gene family (based on at least 96% homology and 80% coverage of the total gene sequence). The list of the 75 genomes is provided in Additional file 5. In a few cases, genes present in multiple copies in some genomes were assigned to the same orthologous family. Thirty-six of these genomes were complete and annotated. For each *Sd*1 draft genome, genes were identified by searching the 6-frame translation of the assembly with protein sequences of a representative for each orthologous gene family. Genes inactivated by non-sense mutations, indels causing a frameshift, or partial deletion were also detected through the 6-frame translation search. ORFs were predicted using Prodigal [52] in regions where no previously known genes were detected. When available, gene annotation from previously annotated genomes was transferred to the new genomes. Genes for which no annotation was available were annotated using Interproscan [53] and search in NCBI's Conserved Domain Database (CDD) [54]. The sequence for these genes is provided in Additional file 9. Antibiotic resistance genes were identified by sequence comparison (blast) with the Antibiotic Resistance Database (ARDB) [55] and potential virulence factors investigated with MvirDB [56]. The COG categories were determined by searching the COG database [57] with rps-blast and selecting hits with an e-value above 0.1. The annotation and distribution of the accessory genes is summarized in Additional file 3.

### Variant detection

The nucleotide sequences of genes belonging to the same gene family were aligned using MUSCLE [58]. Single nucleotide polymorphisms (SNPs) were detected based on these alignments. The SNPs found in the *Sd*1 genomes were verified by realigning the reads on the nucleotide sequences of the genes (and surrounding 100 bp) with BWA and calling variants with SAMtools [49, 50]. All SNPs are listed in Additional file 6. We excluded from this analysis any family that was predicted to include recombined genes or genes found in multiple copies in at least one of the genomes.

### Detection of recombination events

Gene recombination was assessed using Geneconv [59]. To detect recombination events in the four *E. coli* strains and *Shigella* strains, we concatenated the alignments of the 1,859 core genes, in the order observed in *E. coli* K12. Sd197 genome was used to represent all *Sd*1 genomes. The validity of each call was manually examined based on SNPs density, homologies and distribution in the phylogenetic tree.

To detect recombination events specific to a subset of *Sd*1 strains, two alignments were used: 1) the concatenation for each genome of gene sequences ordered as in the genome of *Shigella dysenteriae* type 1 strain Sd197 and 2) the reconstitution of each genome from the alignment of sequence reads to the sequence of Sd197 based on the SNPs and indels. Neither approach predicted recombination events.

### Phylogenetic reconstruction

The phylogenetic tree including 56 *Sd*1 strains, *E. coli* K12, O157, O55, *S. flexneri* and *S. sonnei* was based on a total of 78,266 SNPs extracted from the alignment of 1,859 core genes. The SNPs were concatenated to form a 78,266 bp sequences alignment. Maximum likelihood trees were constructed with Phyml v3.0 [60] using a GTR substitution model and visualized with Dendroscope [61].

The evolutionary relationships among the 56 *Sd*1 strains were investigated using the concatenation of the 919 polymorphic positions found over 689 genes out of the 2,807 core genes (the other core genes were identical in all strains). The best-fit nucleotide substitution model for this data was GTR, as determined with jModelTest 0.1.1 [62]. Maximum likelihood trees were constructed with Phyml v3.0 [60] and visualized with dendroscope [61]. In every case, 1,000 bootstrap repetitions gave values above 900 for most branches.

### Assessment of the role of selective pressures on *Sd1* evolution

Genes from the pan-genome were assigned to a COG category (see Sequence annotation). The proportion of genes in each category versus total number of genes was calculated for the entire pan-genome, genes containing non-synonymous substitutions, and genes that were lost in some strains. The proportions in each category were compared between total pan-genome, genes containing non-synonymous substitutions, and genes that were lost in some strains using a Fisher exact test. All categories showed similar proportions except for lipid metabolism. The strength of selective pressures on the evolution of these genomes was also assessed with the dN/dS ratio for each pair of genomes (56 × 56 comparison). A ratio close to zero indicates that there is a strong selective pressure promoting the conservation of the protein sequence. If the ratio is greater than 1, variants with new protein sequences (and potentially new or altered function) are selected. If the ratio is close to 1, no selective constraint operates on the evolution of the genomes (their evolution is the result of genetic drift). Since most genes have only one variable position, it is impossible to calculate the dN/dS ratio for each gene separately. Instead, for each pair of genomes, we calculated the rate of non-synonymous substitution over all genes (ratio of the total number of non-synonymous substitutions over the total number of possible non-synonymous substitutions) as well as the synonymous substitution rate. The ratio of the overall dN and dS provides an overall estimate of the dN/dS for each pair. Synonymous and nonsynonymous substitution rates were calculated with program yn00 from the PAML version 4.7 package [63] using the method of Yang and Nielsen [64]. All pairwise comparisons yielded a ratio close to 1 (summarized in Additional file 8). Some pairs had a ratio above 1.3; however, this occurred in pairs of very close strains showing little genetic difference, which may artificially inflate the dN/dS ratio.

### Age of most recent common ancestor

The 919 bp “concatenome” was used to assess the age of the most recent common ancestor following a Bayesian approach implemented in the software BEAST v1.7.2 [26]. The software requires setting multiple parameters that define the assumed model of evolution for these genomes. The nucleotide substitution model used was GTR, since prior evaluation for reconstructing phylogenies had identified it as the best model (see Phylogenetic reconstruction). To determine which clock and tree prior was best fitting the data we tested the combination of the following parameters: 1) molecular clock: strict clock, lognormal relaxed clock (uncorrelated), exponential relaxed clock (uncorrelated), and random local clock, 2) tree prior: constant size, exponential growth, logistic growth, Bayesian skyline, Gaussian Markov random field (GMRF) Bayesian Skyride and 3) clock rate prior: CTMC Rate Reference, Gamma, Normal (with default initial values). For each parameter combination, Markov chains of 500 million in length were generated with samples taken every 1,000 MCMC generations. The results of these simulations were compared by their AICM values [27]. The molecular clock was the most influential parameter, since the AICM values tended to be similar for all combinations with the same molecular clock model. The best-fit model for our data was the lognormal relaxed clock model. The best demographic model was the Gaussian Markov random field skyride model [28].

## Availability of supporting data

All the sequencing data generated for this project are available through a bioproject (accession number PRJNA186649) at the National Center for Biotechnology Information (NCBI).

## Electronic supplementary material

Additional file 1: **Details about collection, assembly and genome content for the 56** ***Sd*** **1 strains compared in this study.** (XLSX 18 KB)

Additional file 2: **Geographical distribution of the strains.** The 56 strains were collected in the various parts of the world where the most recent pandemics took place: Africa (light pink), Central America (light orange) and South Asia (light yellow) described in Levine et al. The countries are colored according to the *Sd*1 phylogenetic clade present there. (PDF 1 MB)

Additional file 3: **Accessory genes, their description, their history and distribution across the 56** ***Sd*** **1 genomes.** Genes at least 98% homologous to genes in annotated *E. coli* and/or *Shigella* genome are assigned the locus tag, gene name and product accession number of the gene in one of these annotated genomes. Genes with no homology to already annotated genes where assigned a locus tag starting with SD1PG. (XLSX 205 KB)

Additional file 4: **SNPs identified in pINV protein coding genes in 47** ***Sd*** **1 strains relative to the Sd197 pINV complete sequence.** (XLSX 18 KB)

Additional file 5: **Details of reference** ***Escherichia*** **and** ***Shigella*** **strains included in this study.** (XLSX 55 KB)

Additional file 6: **The 1,859 core genes used to build the phylogenetic tree displayed in Figure** 1 **a.** (XLSX 117 KB)

Additional file 7: **Genes subjected to recombination.** The concatenated 1,859 core genes sequences for the genomes of *Shigella dysenteriae* Sd197, *Shigella sonnei* Ss046, *Shigella flexneri* 2a, *E. coli* UTI89, *E. coli* O157 and *E. coli* K12 were aligned and used with Geneconv to predict which genes were subjected to recombination. For each genome, the predicted recombined are mapped based on the gene index and color-coded based on the predicted sequence donor: 1) one of the analyzed genomes or a close relative 2) an unknown genome donor. The respective number of SNPs due to recombination is indicated in parenthesis next to the name of each genome. (PDF 138 KB)

Additional file 8: **Root to tip distance relative to tip dates.** Distances were generated with Phyml. Points are color-coded by clades (see legend in the figure). (PDF 333 KB)

Additional file 9: **Single nucleotide polymorphisms (SNPs) between the 56** ***Sd*** **1 strains described in Additional file** 1 . (XLSX 270 KB)

Additional file 10: **Results from different BEAST runs (MCMC length of chain = 500,000,000, burnin = 50,000,000), ordered by AICM values.** TMRCA (the most recent common ancestor) is dated in number of years from 2003 (most recent date in the analysis). (XLSX 64 KB)

Additional file 11: **Nucleotide sequence of the novel genes predicted in genomes sequenced for this analysis.** (TXT 231 KB)
