# Supplementary figures and images for: Genomic analysis of the emergence of 20th century epidemic dysentery
Source: BMC Genomics. 2014 May 10;15(1):355. doi: 10.1186/1471-2164-15-355 (PMC4038718; doi:10.1186/1471-2164-15-355)

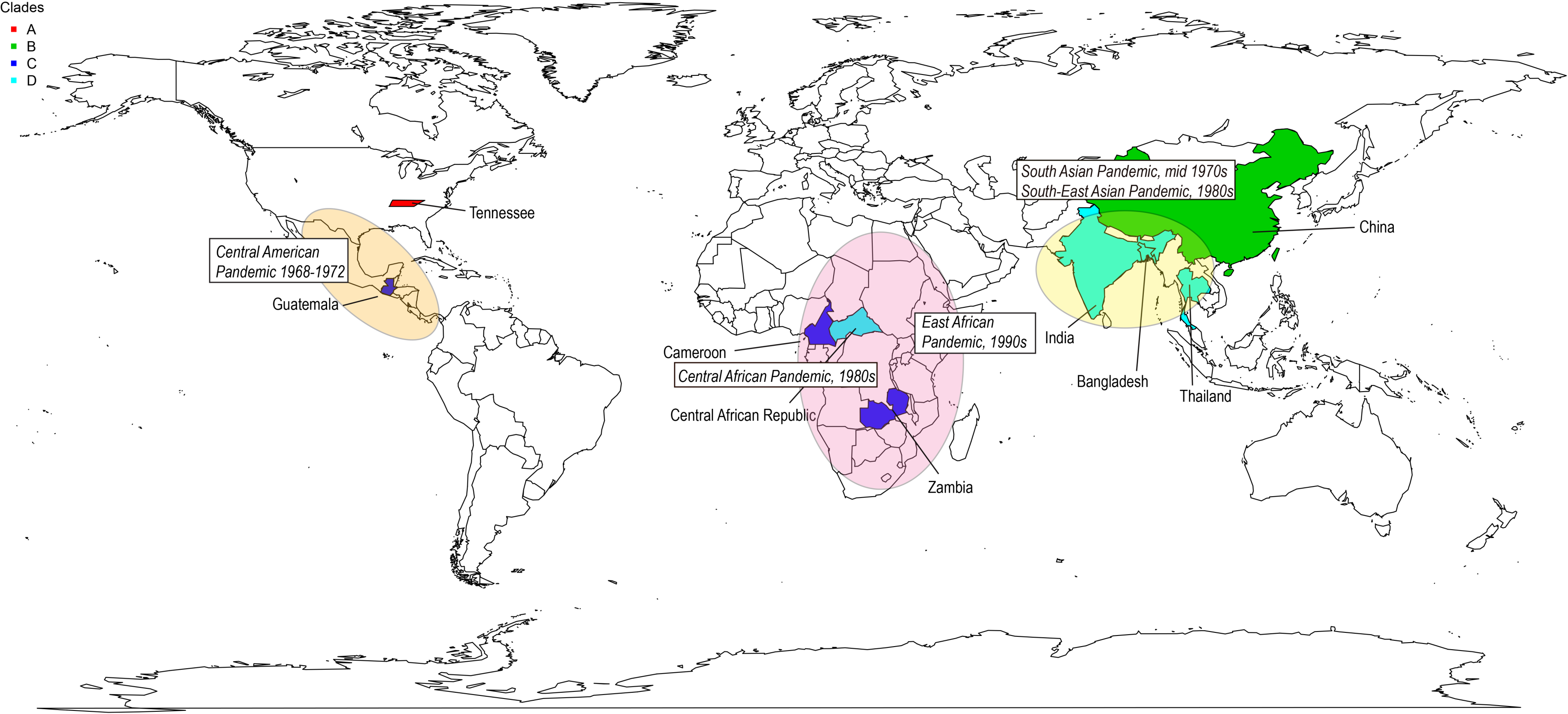

Supplement: Supplementary file 2 — Additional file 2: Geographical distribution of the strains. The 56 strains were collected in the various parts of the world where the most recent pandemics took place: Africa (light pink), Central America (light orange) and South Asia (light yellow) described in Levine et al. The countries are colored according to the Sd1 phylogenetic clade present there. (PDF 1 MB) [file 12864_2013_6073_MOESM2_ESM.pdf]

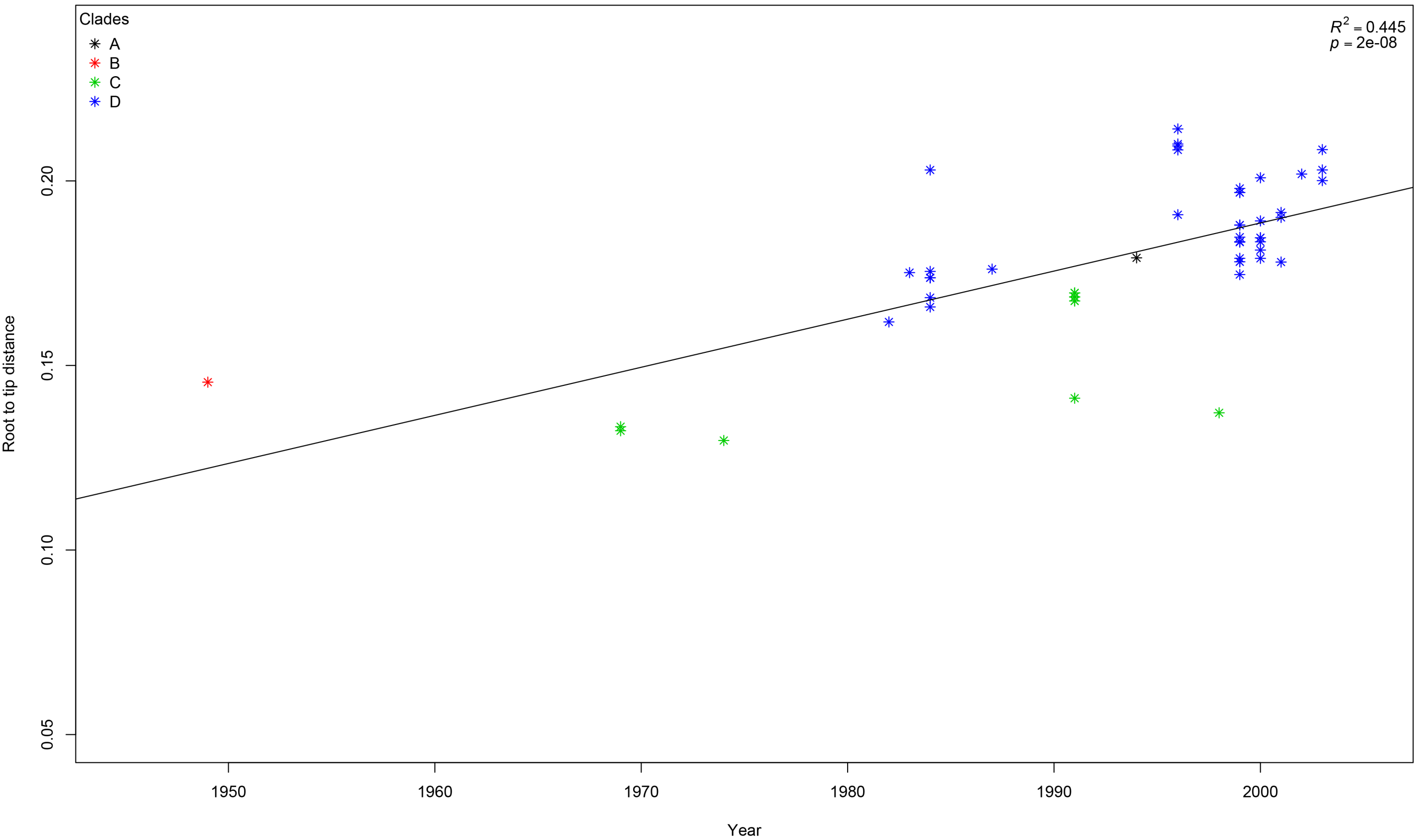

Supplement: Supplementary file 8 — Additional file 8: Root to tip distance relative to tip dates. Distances were generated with Phyml. Points are color-coded by clades (see legend in the figure). (PDF 333 KB) [file 12864_2013_6073_MOESM8_ESM.pdf]
