# Supplementary material for: Genomic analysis of the emergence of 20th century epidemic dysentery
Source: BMC Genomics. 2014 May 10;15(1):355. doi: 10.1186/1471-2164-15-355 (PMC4038718; doi:10.1186/1471-2164-15-355)
Supplement: Supplementary file 7 — Additional file 7: Genes subjected to recombination. The concatenated 1,859 core genes sequences for the genomes of Shigella dysenteriae Sd197, Shigella sonnei Ss046, Shigella flexneri 2a, E. coli UTI89, E. coli O157 and E. coli K12 were aligned and used with Geneconv to predict which genes were subjected to recombination. For each genome, the predicted recombined are mapped based on the gene index and color-coded based on the predicted sequence donor: 1) one of the analyzed genomes or a close relative 2) an unknown genome donor. The respective number of SNPs due to recombination is indicated in parenthesis next to the name of each genome. (PDF 138 KB) [file 12864_2013_6073_MOESM7_ESM.pdf]

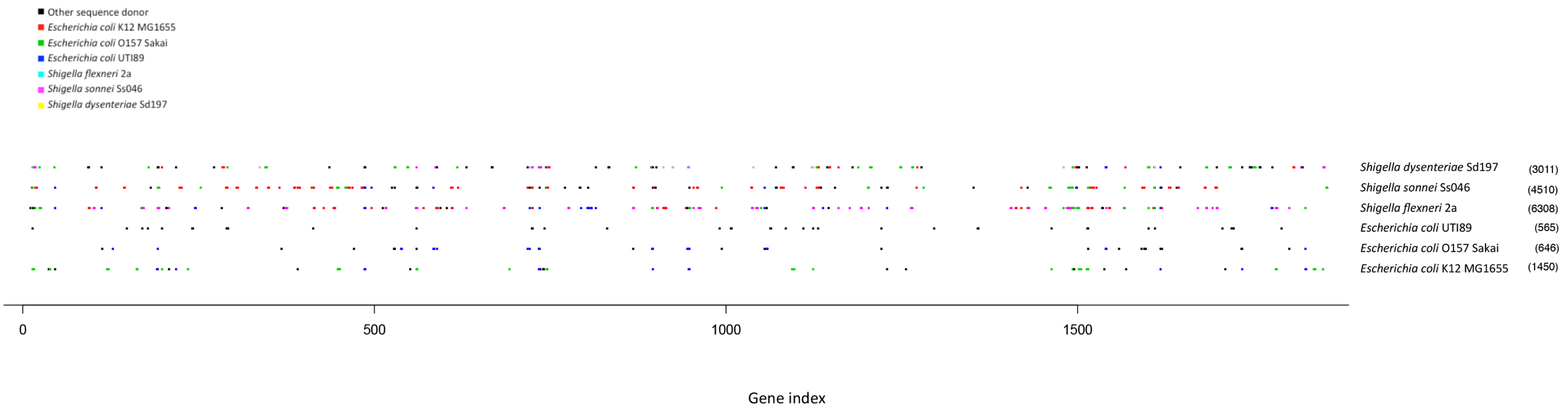

Other sequence donor
Escherichia coli K12 MG1655
Escherichia coli O157 Sakai
Escherichia coli UTI89
Shigella flexneri 2a
Shigella sonnei Ss046
Shigella dysenteriae Sd197
Shigella dysenteriae Sd197 (3011)
Shigella sonnei Ss046 (4510)
Shigella flexneri 2a (6308)
Escherichia coli UTI89 (565)
Escherichia coli O157 Sakai (646)
Escherichia coli K12 MG1655 (1450)
0
500
1000
1500
Gene index
